# EMANCIPATORY PEDAGOGY: PRAXIS FOR RADICAL TRADITIONS OF LEARNING AND HEALING

**DOI:** 10.1093/geroni/igae098.1739

**Published:** 2024-12-31

**Authors:** Nicholas DiCarlo, Brittney Pond

**Affiliations:** Chair; Co-Chair

## Abstract

The Emancipatory Sciences aim to advance knowledge and the realization of dignity, access, equity, healing, and social justice through individual and collective agency and social institutions. Literature suggests this theoretical framework, method, and practice embraces and pursues an impact on research, pedagogy, and systemic change within and beyond academia (Estes, DiCarlo, and Yeh, 2023; Reyes, Versey, and Yeh, 2022). This symposium aims to discuss emancipatory science and critical gerontology through intergenerational, intersectional, and healing oriented frameworks. We address the central questions: who participates and collaborates in such an endeavor and what methodologies and pedagogical approaches strengthen collective participation and democratize access to knowledge across generations. The presenters in this symposium discuss this central question through various facets, including 1) the use of Crone pedagogies to embrace our embodied histories and teach about life course perspectives grounded in standpoint theories of positionality in the classroom 2) UCSF’s Emancipatory Sciences Lab as praxis that creates collaborative spaces for co-learning 3) tangible findings from a digital storytelling project and 4) methodological considerations in participatory action research such as co-design, co-learning, and strengths-based language and design.

